# 5-Flucytosine Longitudinal Antifungal Susceptibility Testing of *Cryptococcus neoformans*: A Substudy of the EnACT Trial Testing Oral Amphotericin

**DOI:** 10.1093/ofid/ofad596

**Published:** 2023-11-29

**Authors:** Thomas C McHale, Andrew Akampurira, Elliot S Gerlach, Atukunda Mucunguzi, Melanie R Nicol, Darlisha A Williams, Kirsten Nielsen, Tihana Bicanic, Ann Fieberg, Biyue Dai, David B Meya, David R Boulware, ENACT Trial Team, Enock Kagimu, Abdu K Musubire, Lillian Tugume, Kenneth Ssebambulidde, John Kasibante, Laura Nsangi, Timothy Mugabi, Jane Gakuru, Sarah Kimuda, Derrick Kasozi, Suzan Namombwe, Isaac Turyasingura, Morris K Rutakingirwa, Edward Mpoza, Enos Kigozi, Conrad Muzoora, Jayne Ellis, Caleb P Skipper, Darlisha A Williams, Kathy H Hullsiek, Mahsa Abassi, Asmus Tukundane, Jane F Ndyetukira, Cynthia Ahimbisibwe, Alisat Sadiq, Florence Kugonza, Shifa Nabbale, Tadeo Kiiza, Alice Namudde, Tony Luggya, Richard Kwizera, Michael Okiror, Dora Babirye, Catherine Nanteza, Susan Mulwana, Rhona Muyise, John Kisembo, Andrew Luswata, Carol Namujju, Eva Laker, Stewart Walukaga, Minda Liu, Nicole Engen, Abduljewad Wele, Irene Rwomushana, Mable Kabahubya, Michael Ssemusu, James Mwesigye, Joan Rukundo, Samuel Jjunju

**Affiliations:** Department of Medicine, University of Minnesota, Minneapolis, Minnesota, USA; Infectious Diseases Institute, Makerere University, Kampala, Uganda; Department of Microbiology & Immunology, University of Minnesota, Minneapolis, Minnesota, USA; Infectious Diseases Institute, Makerere University, Kampala, Uganda; Department of Experimental and Clinical Pharmacology, University of Minnesota, Minneapolis, Minnesota, USA; Department of Medicine, University of Minnesota, Minneapolis, Minnesota, USA; Department of Microbiology & Immunology, University of Minnesota, Minneapolis, Minnesota, USA; Institute of Infection and Immunity, St Georges, University of London, London, UK; Division of Biostatistics, School of Public Health, University of Minnesota, Minneapolis, Minnesota, USA; Division of Biostatistics, School of Public Health, University of Minnesota, Minneapolis, Minnesota, USA; Department of Medicine, University of Minnesota, Minneapolis, Minnesota, USA; Infectious Diseases Institute, Makerere University, Kampala, Uganda; Department of Medicine, University of Minnesota, Minneapolis, Minnesota, USA

**Keywords:** 5-flucytosine resistance, antifungal resistance, antifungal susceptibility testing, cryptococcal meningitis

## Abstract

**Background:**

The EnACT trial was a phase 2 randomized clinical trial conducted in Uganda, which evaluated a novel orally delivered lipid nanocrystal (LNC) amphotericin B in combination with flucytosine for the treatment of cryptococcal meningitis. When flucytosine (5FC) is used as monotherapy in cryptococcosis, 5FC can induce resistant *Cryptococcus* mutants. Oral amphotericin B uses a novel drug delivery mechanism, and we assessed whether resistance to 5FC develops during oral LNC–amphotericin B therapy.

**Methods:**

We enrolled Ugandans with HIV diagnosed with cryptococcal meningitis and who were randomized to receive 5FC and either standard intravenous (IV) amphotericin B or oral LNC–amphotericin B. We used broth microdilution to measure the minimum inhibitory concentration (MIC) of the first and last cryptococcal isolates in each participant. Breakpoints are inferred from 5FC in *Candida albicans*. We measured cerebral spinal fluid (CSF) 5FC concentrations by liquid chromatography and tandem mass spectrometry.

**Results:**

*Cryptococcus* 5FC MIC_50_ was 4 µg/mL, and MIC_90_ was 8 µg/mL. After 2 weeks of therapy, there was no evidence of 5FC resistance developing, defined as a >4-fold change in susceptibility in any *Cryptococcus* isolate tested. The median CSF 5FC concentration to MIC ratio (interquartile range) was 3.0 (1.7–5.5) µg/mL. There was no association between 5FC/MIC ratio and early fungicidal activity of the quantitative rate of CSF yeast clearance (*R*^2^ = 0.004; *P* = .63).

**Conclusions:**

There is no evidence of baseline resistance to 5FC or incident resistance during combination therapy with oral or IV amphotericin B in Uganda. Oral amphotericin B can safely be used in combination with 5FC.

## Graphical Abstract

**Graphical Abstract:**
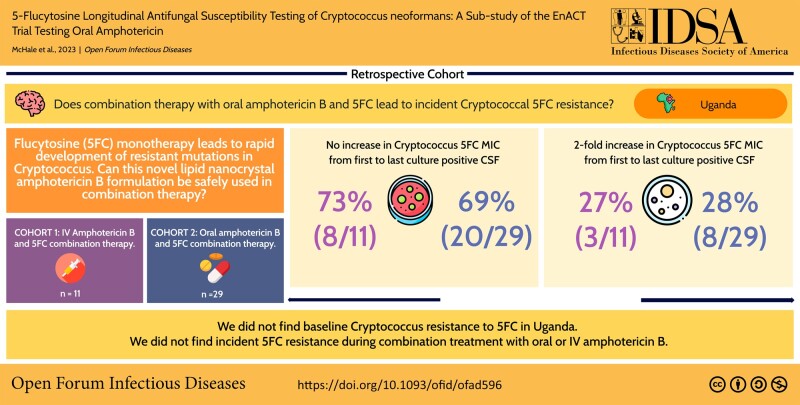
This graphical abstract is also available at Tidbit: https://tidbitapp.io/tidbits/5-flucytosine-longitudinal-antifungal-susceptibility-testing-of-cryptococcus-neoformans-a-sub-study-of-the-enact-trial-testing-oral-amphotericin-d56fad6c-7367-43e3-bd40-38b328652247

Cryptococcal meningitis remains the most common cause of adult meningitis in Africa and is responsible for a disproportionate burden of mortality related to advanced HIV disease globally [1, 2]. Up to 19% of all deaths related to advanced HIV are due to cryptococcal meningitis, which is a consistent finding over recent decades [3, 4]. In addition, the treatment of cryptococcosis is extraordinarily difficult in resource-limited settings, with acute mortality rates ranging from 10% to 20% in resource-rich settings compared with 20%–60% in resource-poor settings [5–7].

In recent years, several developments have led to changing approaches in treating cryptococcal meningitis, especially during the acute phase, known as induction therapy [8, 9]. In 2018, Molloy et al. published the ACTA trial, which systematically evaluated the most commonly used therapies for cryptococcal meningitis [10]. This trial demonstrated that a 1-week regimen of amphotericin B deoxycholate had fewer adverse events than and efficacy similar to a 2-week amphotericin B deoxycholate regimen and that flucytosine (5FC) was superior to fluconazole as adjunctive treatment during induction therapy for cryptococcal meningitis [10]. In 2022, Jarvis et al. published the AMBITION-*cm* trial, which found that a single high dose (10 mg/kg) of liposomal amphotericin B in combination with 5FC and fluconazole has fewer adverse events and similar efficacy compared with standard 1-week amphotericin B deoxycholate plus 5FC [11]. In an effort to continue innovation of cryptococcal meningitis therapeutics, we conducted a phase 2 clinical trial evaluating a novel oral formulation of amphotericin B, known as lipid nanocrystal (LNC) amphotericin B. In mouse models, the experimental oral LNC–amphotericin B with 5FC was found to be equally efficacious compared with injected amphotericin B deoxycholate with 5FC [12]. The phase 2 trial was conducted in Uganda and involved 4 cohorts, which assessed different approaches to oral amphotericin delivery in combination with 5FC [13]. They included intravenous (IV) amphotericin B deoxycholate followed by LNC–amphotericin B, LNC amphotericin B followed by IV amphotericin B deoxycholate, or immediate initiation of LNC–amphotericin B without IV amphotericin B (Supplementary Figure 1).

5FC is a base pyrimidine analog that is converted to 5-fluorouracil (5-FU) intracellularly and inhibits DNA and RNA cellular synthesis [14]. Resistance can develop by mutations that prevent cellular uptake or enzymatic changes that prevent the conversion of 5FC to 5-FU. Amphotericin B and fluconazole have been shown to work synergistically with 5FC, as disrupting cell wall integrity improves 5FC uptake into the cell [15, 16]. To date, there have not been established pharmacokinetic (PK)/pharmacodynamic (PD) index targets for 5FC in *Cryptococcus* [17, 18]. However, we can extrapolate from the use of 5FC in *Candida albicans* that time over minimum inhibitory concentration (MIC) is the key factor in drug efficacy, but target time over MIC remains unknown [18, 19].

While previous studies have shown that 5FC is an essential component of cryptococcal meningitis therapy, use of 5FC as monotherapy can induce stable, highly resistant *Cryptococcus* mutants [10, 20, 21]. As LNC–amphotericin B utilizes a novel formulation and experimental delivery of the amphotericin B molecule, failure of absorption would result in effective 5FC monotherapy. Thus, we assessed cryptococcal isolates from infected participants in the phase 2 trial for development of 5FC resistance during therapy in the control and intervention arms. We evaluated isolates from all participants in the fourth cohort of the trial, in which participants in the experimental arm immediately started oral LNC–amphotericin with 5FC, and the control arm received 7 days of amphotericin B liposomal or deoxycholate and 5FC (Supplementary Figure 1). The majority of controls received liposomal amphotericin B at 3 mg/kg/d.

## METHODS

### Study Design

We conducted a prospective evaluation, nested within the phase 2 clinical trial evaluating oral LNC–amphotericin B (MAT2203) and 5FC combination therapy (ClinicalTrials.gov ID NCT04031833) [13]. The phase 2 trial was a randomized controlled clinical trial. Participants were randomized in a 2.5:1 ratio to the experimental or control arm. All participants had a lumbar puncture performed on the day of enrollment into the study and then on approximately days 3, 7, and 14 and additionally as clinically indicated. Participants in the experimental arm were started on 1.8 g/d LNC–amphotericin B with 100 mg/kg/d 5FC for 2 weeks. Participants randomized to the control group received 3 mg/kg/d IV liposomal amphotericin B (AmBisome, Gilead Sciences, Foster City, CA, USA) or 1.0 mg/kg/d IV amphotericin B deoxycholate plus 100 mg/kg/d flucytosine in 4 divided doses for 7 days then fluconazole 1200 mg/d through 14 days, per World Health Organization cryptococcal guidelines [8].

We followed the European Committee on Antimicrobial Susceptibility Testing (EUCAST) guidelines for yeast susceptibility testing to determine the 5FC MIC [22]. There are no established breakpoints for 5FC in *Cryptococcus*; thus we used epidemiologic cutoff values to infer approximate resistance [23]. We performed susceptibility testing on each participant's first cerebrospinal fluid (CSF) *Cryptococcus* culture isolate and then additionally on the participant's last culture-positive CSF specimen.

### Setting

Participants were enrolled in the trial at Mulago National Referral Hospital or Kiruddu Referral Hospital in Kampala, Uganda, or Mbarara Regional Referral Hospital in Mbarara, Uganda. *Cryptococcus* isolates were cultured and frozen in glycerol for storage along with CSF at −80°C and then transported to the University of Minnesota for 5FC MIC and PK analyses.

### Participants

We enrolled adults with cryptococcal meningitis, diagnosed by CSF cryptococcal antigen lateral flow assay (IMMY, Norman, OK, USA). Participants were excluded if they received >2 doses of IV amphotericin B before enrollment, were unable to take enteral medications, had a Glasgow coma scale (GCS) <15, were deemed unlikely to attend clinic visits, had suspected paradoxical immune reconstitution syndrome, or initiated HIV therapy in the prior 2 weeks. Participants with a prior episode of cryptococcal meningitis who had been treated with 5FC were eligible for inclusion in the study. One participant who was enrolled at the Mbarara study site did not have an isolate available for susceptibility testing. Written informed consent was obtained from participants during the EnACT trial, and the trial was approved by the University of Minnesota Institutional Review Board and local ethics committees in Uganda [13].

### Antifungal Susceptibility Testing

The EUCAST guidelines for yeast susceptibility testing recommended broth microdilution to determine susceptibility [22]. A 96-well, flat-bottom, nontreated tissue culture plate was prepared with 100 µL of RPMI 1640 broth media and 5FC serial dilutions ranging from 256 to 0.5 µg/mL. *Cryptococcus neoformans* cells were prepared from a −80°C glycerol stock. After washing and resuspending in sterile water, a 100-µL inoculum with a final cell concentration of ∼10^5^ cells/mL was added in triplicate to the 96-well plate for each concentration of 5FC and incubated at 37°C for 72 hours. The degree of growth was quantified with light spectrophotometry at an optical density of 600 nm. Triplicate wells were averaged together, and the 5FC MIC was determined to be the lowest 5FC concentration that gives ≥50% inhibition of growth compared with the drug-free controls.

### Quantitative Flucytosine Concentration Measurement

Following protein precipitation from CSF with acetonitrile, 5FC quantitative CSF measurements were performed using high-performance liquid chromatography (Agilent 1100 Series, Santa Clara, CA, USA) coupled with a Sciex API4000 triple quadrupole instrument (MDS-SCIEX, Concord, ON, Canada). Mass spectrometric detection was performed using multiple reaction monitoring in positive ionization mode. The assay calibration range was 0.01–100 µg/mL. Quality control and calibration standards were prepared with bovine CSF, and 5FC 13C was used as the internal standard for all samples.

As we did not have access to the exact timing of 5FC dosing, which was given in 4 divided doses, and lumbar punctures were performed at variable timing, we used the average 5FC CSF concentration over the course of 5FC treatment to determine approximate concentrations. To calculate the 5FC/MIC ratio, all 5FC CSF concentrations obtained while the participant was receiving 5FC were averaged (ie, CSF collected over days 3–14 for the LNC–amphotericin B group and days 3–7 for the control group). This provides a rough proxy for time over MIC, which could not be calculated given the limitations of our data.

### Statistical Methods

To investigate potential differences in susceptibility between the control and LNC–amphotericin B arms, we utilized the chi-square test to compare observed 5FC MICs. The chi-square test compares the observed frequencies for each MIC category (serial dilution of 5FC) with the frequencies that would be expected if there was no association between the treatment groups. As only 1 participant had a 4-fold change in susceptibility in the entire cohort, we did not perform a statistical analysis on the 5FC MIC change over time between groups. We also analyzed the relationship between CSF early fungicidal activity (EFA) and 5FC MIC or 5FC/MIC ratio using linear regression with 5FC MIC or 5FC/MIC ratio as the dependent variable and EFA as the independent variable. EFA is a surrogate marker for mortality and a commonly used end point in phase 2 trials for cryptococcal therapeutics [24]. For EFA, we calculated linear models separately for the control and LNC–amphotericin B groups. We performed all statistical analyses and generated figures using R (R Core Team, 2021) [25].

## RESULTS

**Figure 1.**
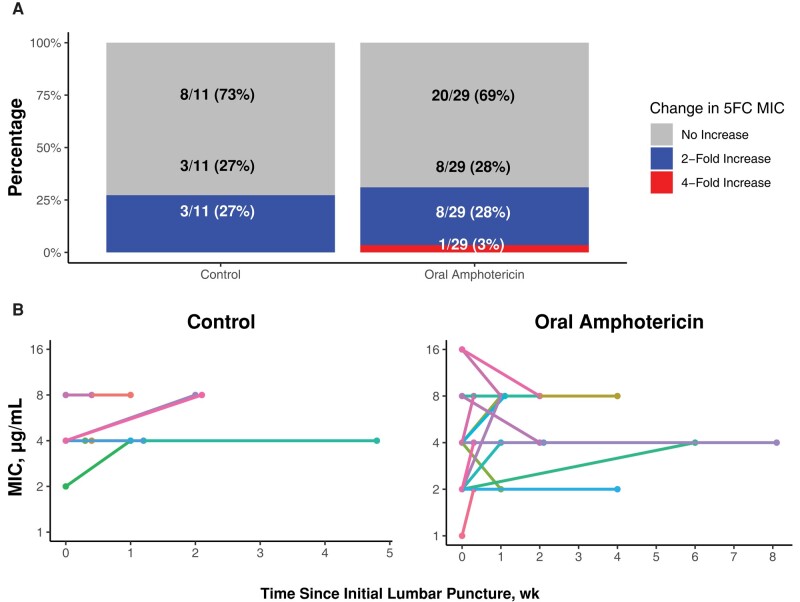
Change in flucytosine susceptibility between first and last *Cryptococcus* isolates. There was minimal change in 5FC MIC between the first and last positive lumbar punctures. *A*, The percentage of participants who had a 2-fold or 4-fold increase between the first and last positive lumbar punctures. *B*, Individual lines representing participants, with the 5FC MIC represented by the dot at each time point, with time displayed on the x-axis. Abbreviations: 5FC, flucytosine; MIC, minimum inhibitory concentration.

In our nested cohort study, 56 participants were enrolled and randomized with 40 participants in the LNC–amphotericin B arm and 16 in the control arm. Overall, 32 participants in the oral LNC–amphotericin B arm and 14 in the control arm grew *Cryptococcus neoformans* on their initial CSF culture. Twenty-nine participants receiving LNC–amphotericin B and 11 controls had at least a second CSF with positive *Cryptococcus* growth. The median time to last culture-positive CSF was 7 days, the minimum time was 1 day, and the maximum time was 50 days (Figure 1). All participants had HIV, the median age was 37 years, 30% were female, and the median weight was 50 kg (Table 1).

**Table 1.** Baseline Characteristics for the HIV+ Cryptococcal Meningitis Study Cohort

*Characteristic*	*Oral LNC*–*Amphotericin B* (n *=* *40*)	*IV Amphotericin B Control* (n *=* *16*)	*Overall* (n *=* *56*)
Age, y	37 [32–43]	37 [31–41]	37 [32–43]
Female	18 (45)	12 (75)	30 (54)
Weight, kg	50.5 [45–55]	50 [45–54.5]	50 [45–55]
CSF results			
Opening pressure, cmH_2_O	13 [6–27]	22 [14–30]	15 [6.5–29]
Culture log_10_ CFU/mL	3.58 [1.78–4.75]	3.33 [1.98–4.43]	3.57 [1.84–4.70]
Culture CFU/mL	3800 [60–56 200]	2140 [95–26 900]	3720 [69–50 100]
Sterile	7 (17.5)	2 (12.5)	9 (16)

Values are median [IQR] or No. (%).

Abbreviations: CFU, colony-forming units; CSF, cerebrospinal fluid; IQR, interquartile range; IV, intravenous; LNC, lipid nanocrystal.

The overall median 5FC MIC (MIC_50_) was 4 µg/mL, and the 90th percentile (MIC_90_) was 8 µg/mL. At the last positive CSF culture, 64% (7/11) of those in the control group had a 5FC MIC of 4 µg/mL, and 36% (4/11) had a 5FC MIC of 8 µg/mL, as compared with 40% (12/29) of those in the oral LNC–amphotericin B group having a 5FC MIC of 4 µg/mL and 50% (15/29) having a 5FC MIC of 8 µg/mL (Table 2). Despite the oral LNC–amphotericin group having some baseline isolates with a 5FC MIC of 16 µg/mL, the 5FC MIC_50_ was the same in both groups. There was no statistical difference in 5FC MICs between the control and oral LNC–amphotericin B groups at first or last lumbar puncture in participants with a positive *Cryptococcus* culture (χ^2^  *P* = .317 and .451, respectively).

**Table 2.** MIC for Flucytosine in ***Cryptococcus*** Isolates From Participants in the Phase 2 Clinical Trial

*MIC*, *µg/mL*	*Control* *IV Amphotericin B*, No. *(%)*	*Oral LNC*– *Amphotericin B*, No. *(%)*	*Total*, No. *(%)*	*P Value*
First lumbar puncture *Cryptococcus* isolate				
≤2	2 (14.3)	7 (21.9)	9 (19.6)	.317
4	9 (64.3)	15 (46.9)	24 (52.2)	
8	3 (21.4)	6 (18.8)	9 (19.6)	
16	0 (0)	4 (12.5)	4 (8.7)	
Last positive lumbar puncture *Cryptococcus* isolate				
≤2	0 (0)	3 (10.3)	3 (7.5)	.451
4	7 (63.6)	12 (41.4)	19 (47.5)	
8	4 (36.4)	14 (48.3)	18 (45.0)	

The top section contains MIC values for the initial diagnostic CSF culture, before 5FC exposure. The lower section contains final CSF culture with positive *Cryptococcus* growth during 18 weeks of follow-up. The chi-square *P* value treats MIC as a categorical variable and tests for a statistical difference in the distribution of MICs between the control and oral LNC–amphotericin B groups.

Abbreviations: 5FC, flucytosine; CSF, cerebrospinal fluid; IV, intravenous; LNC, lipid nanocrystal; MIC, minimum inhibitory concentration.

**Figure 2.**
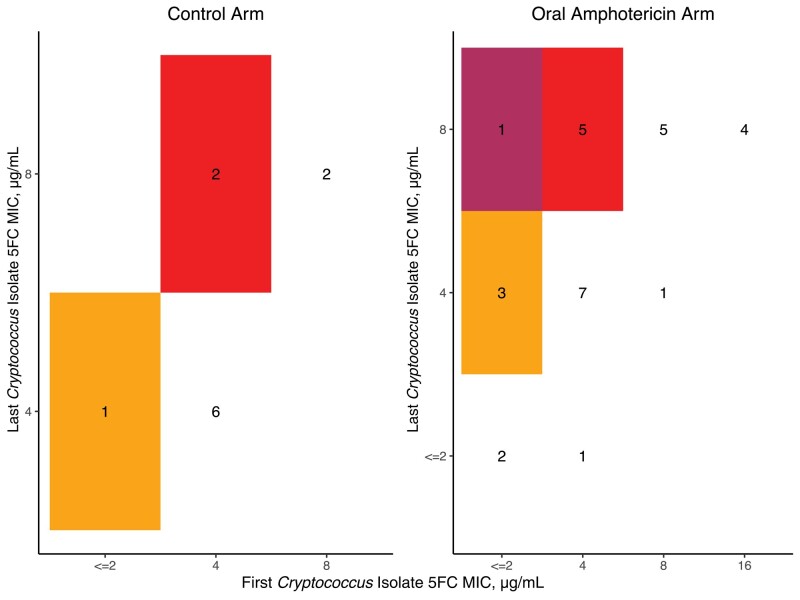
Checkerboard of flucytosine susceptibility between the first and last *Cryptococcus* isolates. In both arms, most participants had a screening and last LP MIC of 4 µg/mL. One participant in the oral amphotericin B arm moved from an MIC of 2 to 8 µg/mL (indicated in the darkest, maroon color shading). Those without a second isolate are recorded as no change. Abbreviations: 5FC, flucytosine; LP, lumbar puncture; MIC, minimum inhibitory concentration.

Overall, change in 5FC MIC between the first and last positive *Cryptococcus* isolates for each individual participant was rare (Figure 1). In the control arm, 73% (8/11) had no change or a decrease in 5FC MIC, while 27% (3/11) of participants had a 2-fold increase in 5FC MIC between the first and last positive *Cryptococcus* isolates. In the oral LNC–amphotericin B arm, 69% (20/29) had no change or a decrease in 5FC MIC, 28% (8/29) of participants had a 2-fold increase in 5FC MIC, and 1 participant (3%) had a 4-fold increase in 5FC MIC between the first and last positive *Cryptococcus* isolates (Figure 2). The individual with a 4-fold increase also responded well to therapy (EFA = 0.301 log_10_ colony-forming units [CFU]/mL/d), with achievement of CSF culture sterility by day 14 and no relapse through 18 weeks of follow-up (Supplementary Figure 2). There were 5 participants who had a positive CSF *Cryptococcus* culture at 4 weeks or beyond (1 in the control group and 4 in the oral amphotericin B group). For these individuals, the median 5FC MIC (range) was 4 (2–8) µg/mL.

**Figure 3.**
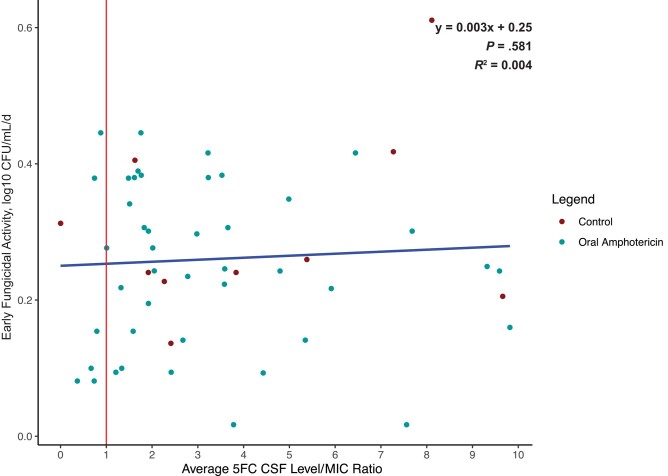
Scatterplot depicting the ratio of average 5FC CSF concentration to MIC. The median 5FC to MIC ratio was 3.0, and the mean was 4.7, indicating that the 5FC CSF concentrations were markedly higher than the MIC in the overall population. Points left of the vertical red line indicate 5FC concentrations below the MIC at any point during treatment. There was no relationship between rate of CSF *Cryptococcus* yeast clearance and the average 5FC CSF concentration to MIC ratio. The blue trendline is based on a linear regression (*R*^2^ = 0.004). Eleven outliers with EFA >0.6 or 5FC/MIC ratio >10 were removed. Abbreviations: 5FC, flucytosine; CSF, cerebrospinal fluid; EFA, early fungicidal activity; MIC, minimum inhibitory concentration.

CSF 5FC concentrations were measured in 52 of 56 participants, generally at day 3 and day 7 of antifungal therapy, as well as day 14 for the oral LNC–amphotericin arm. The median intraparticipant 5FC CSF average concentration (interquartile range [IQR]) was 15.1 (11.9–20.0) µg/mL. The median 5FC/MIC ratio (IQR) was 3.0 (1.7–5.5) µg/mL (Figure 3). The average CSF 5FC concentration was ≥8 µg/mL for 90% of participants and ≥16 µg/mL for 36% of participants.

**Figure 4.**
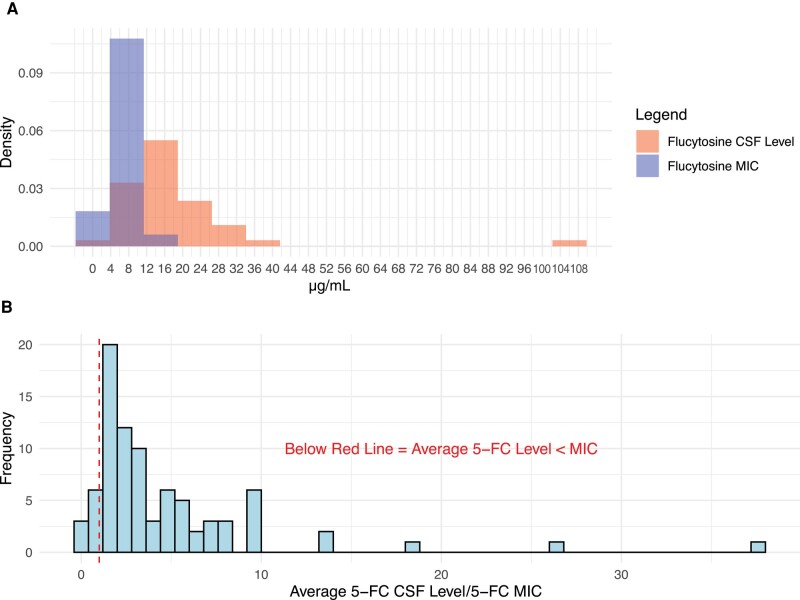
Density plot of average flucytosine (5FC) CSF concentration to *Cryptococcus* MIC. The average 5FC CSF concentration was rarely less than the MIC for any individual participant. *A*, Minimal overlap in a density plot of 5FC CSF concentrations and MIC. *B*, Ratio of 5FC CSF concentrations to 5FC MIC for each participant. A ratio of <1.0 would indicate CSF concentrations of 5FC are below than MIC, which occurred in 13.3% (n = 6) of participants. Abbreviations: 5FC, flucytosine; CSF, cerebrospinal fluid; EFA, early fungicidal activity; MIC, minimum inhibitory concentration.

We assessed whether the early fungicidal CSF clearance rate of *Cryptococcus* was associated with *Cryptococcus* 5FC MIC, 5FC CSF concentration, or 5FC/MIC ratio. We found no statistical association between EFA and 5FC MIC in the control group or the LNC–amphotericin B group (Figure 3). The linear regression of 5FC MIC and EFA has a ß coefficient of −0.523 log_10_ CFU/mL CSF/d per µg/mL MIC (*P* = .76) for the control group and −0.373 log_10_ CFU/mL CSF/d per µg/mL MIC (*P* = .68) for the oral LNC–amphotericin B group (Supplementary Figure 3). 5FC CSF concentrations were measured for each CSF sample provided and averaged per individual participant. There was no association between 5FC/MIC ratio and rate of CSF clearance (*R*^2^ = .004; *P* = .63). Six participants had average 5FC concentrations that were below the 5FC MIC at any point during treatment, of which only 2 participants were consistently below the MIC, and all had an MIC change of ≤2-fold during treatment (Figures 3 and 4).

## DISCUSSION

We observed no clinically significant change in the *Cryptococcus* susceptibility to flucytosine during the course of induction therapy with either standard-of-care IV amphotericin B or experimental oral LNC–amphotericin B. With IV amphotericin B, no participants had a 4-fold change in MIC during the treatment course, and in the oral LNC–amphotericin B arm, 3% (1/29) had a 4-fold increase in 5FC MIC. Importantly, the participant who had a 4-fold change in MIC still responded appropriately to therapy. Given the variability in laboratory techniques and the heterogeneity of *Cryptococcus* clinical isolates upon passage, a 2-fold change in MIC is generally considered natural variation, as opposed to a true change in molecular physiology [26, 27]. We also found that no baseline resistance to 5FC was present in *Cryptococcus* isolates in Uganda. This is likely because 5FC is not used in agriculture as a fungicide, due to the same problem that monotherapy rapidly drives resistance.

The median 5FC intraperson average CSF concentration observed (IQR) was 15.1 (11.9–20.0) µg/mL, which is slightly lower, but generally within range of previously reported values [17, 18]. For example, in an evaluation of 5FC CSF concentrations during the AMBITION clinical trial, Stott et al. predicted a minimum median 5FC CSF concentration (IQR) of 23.9 (15.3–43.5) µg/mL [11, 18]. The 5FC CSF concentrations may have been lower in our cohort due to the use of oral amphotericin B, which was associated with higher rates of nausea and vomiting and may have led to decreased drug exposure [13]. Finally, we observed that the average 5FC CSF concentrations were below the 5FC MIC at any point during treatment in 13.3% (n = 6) of participants, and none of these had a significant change in 5FC MIC during the treatment course.

One method of identifying resistance is to use epidemiological cutoff values, which indicate the general distribution of MIC values in a population and identify MICs where resistance genes are likely to be found. In a large meta-analysis that identified 5FC MICs for >3000 clinical isolates, ≥95% of isolates had an MIC <16 µg/mL [23]. The largest survey of MICs to date of 164 clinical isolates found that the prevalence of 5FC MIC ≥32 µg/mL for *Cryptococcus* was 1%–2% globally [28]. Based on these data, we considered an MIC of ≤4 µg/mL to be susceptible, 8–16 µg/mL to be intermediate, and ≥32 µg/mL to be resistant. We found that 63% (n = 55, *Cryptococcus* isolates) were fully susceptible (MIC ≤ 4 µg/mL) and 36.8% (n = 32, *Cryptococcus* isolates) were intermediate (8 ≤ MIC ≤ 16 µg/mL), based on this arbitrary classification scheme. The quantitative EFA did not differ across MICs. The lack of association may be due to the fact that we did not have any significant 5FC drug resistance in the population, and therefore we were able to achieve effective levels of 5FC even in those participants with a higher MIC. Thus we would recommend not measuring 5FC MIC in clinical practice and continuing to give 5FC as part of combination antifungal therapy, regardless of the in vitro MIC measurement.

Resistance to 5FC is an important yet understudied concept in cryptococcal meningitis management. Previous studies have demonstrated that monotherapy with 5FC induces stable, highly resistant *Cryptococcus* mutants [20, 29, 30]. For this reason, 5FC is always administered in combination with fluconazole or amphotericin B [10, 20]. However, the clinical implications of resistance remain poorly understood. Studies suggest that fluconazole resistance may be responsible for *Cryptococcus* relapse or poor response to therapy [31, 32]. In a systematic review of fluconazole resistance, the incident isolate had a fluconzole resistance rate of 10.6% compared with 24.1% in relapse isolates, indicating possible treatment-emergent resistance [33]. However, the role of 5FC resistance in poor treatment response and relapse is not well understood. This is the first study, to our knowledge in humans, to evaluate the dynamic changes to 5FC MIC over a 2-week combination treatment course. We also establish that emergent resistance to 5FC is unlikely to occur within this timeframe in the context of amphotericin B combination treatment, and this remains true when using an experimental orally available amphotericin B. While our study enrolled participants with a normal GCS, this is unlikely to be associated with higher fungal burdens, as a previous study linked altered mental status to immune response rather than fungal burden [34]. Furthermore, higher fungal burdens would not be expected to be a risk for developing resistance if oral LNC–amphotericin B were an effective therapy. Within our cohort, participants with higher fungal burdens (>50 000 CFU/mL) were not more likely to develop 5FC resistance or have a change in MIC during the course of therapy (Supplementary Figure 4). Therefore, we believe that these results are generalizable even to patients with more severe forms of cryptococcal meningitis.

The lack of an association between 5FC MIC and EFA suggests that even subjects with *Cryptococcus* isolates in the “intermediate resistant” range have good outcomes. These data suggest that *Cryptococcus* has less natural resistance to 5FC, as shown by the low MIC, when compared with fluconazole, which has been shown previously to exhibit higher MICs that have been increasing over time with agricultural azole usage [35, 36].

Our study is primarily limited by the moderate number of isolates tested for 5FC susceptibility. The fact that we did not identify any fully resistant isolates suggests that we did not accumulate enough participants to identify the expected 1%–2% of isolates that are resistant to 5FC. EFA is a surrogate marker that can predict mortality when it meets certain thresholds [24]. For example, an EFA <0.2 log_10_ CFU/d indicates increased mortality, and EFA >0.3 log_10_ CFU/d predicts improved mortality [24]. Given its widespread use in phase 2 clinical trials for *Cryptococcus* therapeutics, EFA is a good direct marker of antifungal activity in humans at the site of infection, which enables one to investigate the clinical implications of MIC differences in our groups. A larger study that accumulated higher numbers of participants and MIC samples would more adequately demonstrate the distribution of MICs in the population and the effect of treatment over time.

As we have discussed, 5FC is an essential component of *Cryptococcus* therapy, but resistance can develop rapidly when it is used as monotherapy. However, the clinical implications of in vitro resistance are still unclear. For example, amphotericin B has been shown to have synergy with 5FC-resistant strains, lowering the in vitro MIC from highly resistant to highly susceptible when tested together [37]. Jezewski et al. also recently tested 5FC susceptibility with higher concentrations of CO_2_, which induced lower in vitro MICs compared with ambient environments [38]. This suggests that the behavior of 5FC in host-like conditions may be different from the behavior in vitro. For these reasons, further study into the clinical implications of resistance is necessary to understand how 5FC resistance should be interpreted and managed. Flucytosine susceptibility should likely not be measured in routine care.

As oral LNC–amphotericin B uses a novel delivery mechanism for oral bioavailability, there was concern that *Cryptococcus* resistance to 5FC could develop if the oral LNC–amphotericin B was ineffective, and 5FC monotherapy was thereby given. Our study suggests that while 5FC CSF levels are slightly lower than previous reports, incident resistance during treatment does not occur.

## Supplementary Material

ofad596_Supplementary_Data

## Supplementary Data

Supplementary materials are available at *Open Forum Infectious Diseases* online. Consisting of data provided by the authors to benefit the reader, the posted materials are not copyedited and are the sole responsibility of the authors, so questions or comments should be addressed to the corresponding author.
