# Supplementary material for: 5-Flucytosine Longitudinal Antifungal Susceptibility Testing of Cryptococcus neoformans: A Substudy of the EnACT Trial Testing Oral Amphotericin
Source: Open Forum Infect Dis. 2023 Nov 29;10(12):ofad596. doi: 10.1093/ofid/ofad596 (PMC10745249; doi:10.1093/ofid/ofad596)
Supplement: ofad596_Supplementary_Data [file ofad596_supplementary_data.zip › 5FC-MIC_supplementary-materials_OFID.docx]

**Supplemental Methods**

**Antifungal Susceptibility Testing**

*Cryptococcus neoformans* cells were streaked from the -80ºC glycerol stock onto a yeast extract peptone-dextrose (YPD) agar plate and incubated for 48 hours at 37ºC. After 48 hours of incubation, cells from the lawn were used to inoculate a YPD broth culture, which was incubated overnight at 37ºC. The cells were washed three times by centrifugation at 3,000 rpm for 15 minutes, the supernatant was removed and then cells were resuspended in sterile water. A hemacytometer was used to prepare a final cell concentration of approximately 10^5^ cells/mL. A 96-well flat bottom, non-treated tissue culture plate with RPMI 1640 broth media was prepared concurrently that contained 5FC serial dilutions ranging from 256 to 0.5 µg/mL. 100µL of media was added to each well and a cell concentration of approximately 1 x 10^5^ cells/mL at a volume of 100µL was inoculated in triplicate wells of the prepared 96-well plate for each 5FC dilution. The plate was prepared with a negative control of deionized water and the positive control of the *Cryptococcus neoformans* strain KN-99$\alpha$. The plates were then incubated at 37ºC with ambient atmospheric conditions for 72 hours. Finally, the amount of fungal growth was quantified at an optical density of 600nm.

**Quantitative Flucytosine Concentration Measurement**

The chromatographic separation was performed with a Phenomenex Synergi Polar-RP (20 x 75 mm), reversed phase column with a 4-micron particle size. The mobile phase used for the gradient elution consisted of (A) 0.1% formic acid in DI water (B) 0.1% formic acid in acetonitrile. The chromatographic conditions were isocratic from 0 to 0.50 min at 0% B, followed by a linear gradient at 0.6 to 2.0 min of 0% - 75% B and returned to the starting conditions at 2.1 minutes, with a flow rate of 0.5 mL/min, for a total run time of 6 minutes. The column temperature was maintained at 30°C.

**Supplemental Figures**

**Supplemental Figure 1: EnACT Cohort 4 Trial Design.** Enrollment for the phase 2 clinical trial was divided into 4 cohorts. Cohorts 1-3 explored various sequences of IV and LNC amphotericin B. Our study took place in the 4^th^ EnACT cohort, which enrolled participants in the experimental arm to receive oral LNC-amphotericin B immediately upon randomization without any IV amphotericin B. Adapted with permission from EnACT phase 2 clinical trial (13).

**Supplemental Figure 2:** The participant in the oral LNC-amphotericin B who had a 4-fold change in MIC (2 → 8 μg/mL) responded appropriately to induction antifungal therapy with a standard expected decrease in CSF quantitative cryptococcal cultures over time with culture sterility achieved by day 14. The EFA for this participant was 0.301 log_10_ *Cryptococcus* CFU/mL CSF/day.

**Supplemental Figure 3:** EFA and MIC are not associated with either the control or LNC amphotericin B groups. The beta coefficient for the control group is -0.523 log_10_ *Cryptococcus* CFU/mL CSF/day per µg/mL MIC (p-value = 0.763) while the beta coefficient for the LNC amphotericin group is -0.373 log_10_ *Cryptococcus* CFU/mL CSF/day per µg/mL MIC (p-value = 0.676).

**Supplemental Figure 4:** Participants with quantitative Cryptococcus cultures >50,000 CFU/mL were not more likely to have a change in 5FC MIC during the course of therapy compared to those with quantitative Cryptococcus culture < 50,000 CFU/mL in both control and oral LNC amphotericin groups. This indicates that participants with higher fungal burdens were not more likely to develop resistance compared to those with lower fungal burdens.
